# Letter from A. W. Bosworth, Paris

**Published:** 1870-05

**Authors:** A. W. Bosworth

**Affiliations:** Paris


PARIS, March 11, 1870.

In the service of Prof. Gosselin, at the Hospital of the Charity, has been, for the last six months, a man about 40 years of age, who had the whole of his right leg severely burned about two years ago; for which, at that time, he entered a hospital, and remained for six months. There being left a wound, about five inches long and one wide, which would not heal, he was then sent to Vincennes, where there is a hospital for men, convalescent, from all the hospitals of Paris. Having remained there a month or six weeks, and feeling strong, he resolved to leave the hospital and begin work. The little wound, still remaining open, now began to enlarge; and finally became so troublesome, that the patient sought aid from Prof. Gosselin. This celebrated surgeon, from the beginning of last October till the middle of March, tried everything possible to make the wound heal. He finally gave up in despair, telling the patient he must leave the hospital, as he could do nothing for him. One of the Internes of the service then proposed to try the autoplastic method, but in a new way.

This latter consists in taking, with a lancet, little pieces of the patient's skin, or of another person's, and putting them on to the wound, where they soon begin to granulate, and around them the skin begins to grow, forming, as it were, little islands of true skin in the midst of the wound. The pieces of skin taken are not larger than the heads of two or three pins taken together, and just thick enough to cause a little bleeding of the part from which they are taken. I examined this patient's leg this morning, and found that the large exposed surface is nearly covered with true skin; and in a very short time he will be completely well, and out of the service. The patient said he felt nothing in the wound. All goes perfectly well.

In doing this little operation, which is certainly much to be recommended, care must be taken that the cut surface of the skin be placed on the surface of the wound, and there maintained with a strip of diachylum till it has formed an union with the part. The ordinary dressing may be placed over the adhesive strip of plaster and rest of the wound.

The Interne, who appears to be the originator of this process, informed me that he had healed in this manner several wounds which had been considered hopeless. Prof. Gosselin has given him three or four patients in his private practice to treat, on whom he had been long uselessly trying to heal their wounds.

I saw, two days ago, Prof. Richet ordering the same treatment for one of these indefinite wounds, result of a burn. He, also, has been trying for months to make the wound heal, and at last gave it up. Being told of this new treatment, he resolved to try it.

It is almost needless to state, that before applying these little pieces of borrowed skin, the wound should first be brought into as healthy a condition as possible; that it would be useless to apply them while the suppuration is very profuse.

As I think of nothing new in the hospitals, allow me to continue with news from the Learned Societies of Paris. At one, M. Onimus presented three dogs, fed alike, and as similar as possible, before beginning his experiments. Two of the dogs were submitted to daily electrical shocks; the third not. The latter, after a short period, was neither so strong nor heavy as the two former; from which he concluded that electricity has a very favorable influence on development and nutrition, and declares the ascending current to be more effective than the descending. These statements led M. Brown Sequard to remark that he had cited, in 1848, observations which proved that nutrition augments rapidly under the influence of galvanism. By-the-way, Dr. Brown Sequard has been obliged to discontinue his lectures at the Faculty on account of ill health. M. Onimus also said, at the same Medical Society, that, in regard to the two electrical currents, it had been observed, in a physiological point of view, that the continued current has on non-striated fibers a far more powerful action than the currents of induction; thus, the continued currents excite contractions of the non-striated fibers of the intestines, and of the bladder. This, he says, leads one to suppose that, by paralysis, the striated muscular element passed to the state of non-striated fibers. We see, from this, that the two kinds of currents have quite different properties. That, in a theoretical point of view, they may serve as a means of diagnosis, and as means of treatment.

The continued currents permit one to act on the nutrition of paralyzed muscles, especially in cases of facial paralysis, where the use of the currents of induction is quite reserved on account of their influence on the optic nerve. If on a paralyzed muscle no effect is obtained by the current of induction, but a contraction be produced by a continued current, we are to conclude that the nerve, in this case, is primarily affected. This is corroborated by physiological experiments.

Every time that the nerve is first affected in any pathological state, the continued currents have a decided effect. If we have to do with pure muscular atrophy, the currents of induction, on the contrary, should be employed.

The Academy of Medicine is now occupied with the utility and noxiousness of flies. Many interesting facts are brought forth. Among the many experimenters on this part of the animal kingdom, M. Davaine says that he has clearly proved that flies can inoculate the malignant pustule three days after having sucked the blood of an animal affected with this disease; that he is not yet prepared to declare that three days is the extreme limit.

Probably you are well aware that for a long time the Academy was occupied with the important question of the mortality of infants. It appears there are 53,000 annually born in Paris; that 25,000 of them are sent out to be nursed, and at the end of every year 12,000 or 13,000 of these little Parisians are dead. This, you see, is an enormous per cent. of deaths; and as the Paris physicians are well aware that in Norway, and certain Departments of France, this same mortality is only ten per cent., it is well they should get a little excited over the question.

It also appears, from the report of the Commission, that there are an immense number of women in Paris who conceive, and bring forth children, yet are unable to suckle them on account of the lactiferous organs not being sufficiently active.

M. Piorry, in the discussion, said that, besides the bad alimentation, we must include change of air, and the journey these little ones are obliged to make at so early a moment. He says the infant should not be given to a nurse before the end of fifteen days, thus allowing the child to be more capable of digesting foreign milk, and of supporting the change of temperature, etc. He, moreover, thinks such a proceeding would tend to diminish the accidents of puerperality.

He demands that the peasant women, who are the great majority of the nurses in Paris, should be compelled to receive a certain amount of instruction in regard to their duties, their rights, morality, and hygiene. It is well known — I have seen it myself — that the great majority of Paris nurses are frightfully ignorant; knowing just enough to give their breast to the child, to wash, dress, and carry it about. French infancy is truly cradled — nursed in ignorance.

I find it not astonishing that so many of these little Parisians are born so soon to die, but that even the comparative few survive such an ocean of dangers. Well is it that the Academy have been so long occupied with this vital question; yet sad it is, too, that they have only revived the subject to send it back to the Commission, that a new report may be made. A certain lapse of time must thus pass, when it will be again brought up, for the Academy again to display its well-known oratory.

Two points worthy of notice in the report of the Commission are, that they recommend a change in the Military Institutions of France, and laws against seduction.

A. W. Bosworth.

